# Diabetes and infectious disease mortality in Mexico City

**DOI:** 10.1136/bmjdrc-2022-003199

**Published:** 2023-03-08

**Authors:** Fiona Bragg, Pablo Kuri-Morales, Jaime Berumen, Adrián Garcilazo-Ávila, Carlos Gonzáles-Carballo, Raúl Ramírez-Reyes, Rogelio Santacruz-Benitez, Diego Aguilar-Ramirez, Louisa Gnatiuc Friedrichs, William G Herrington, Michael Hill, Eirini Trichia, Rachel Wade, Rory Collins, Richard Peto, Jonathan R Emberson, Jesus Alegre-Diaz, Roberto Tapia-Conyer

**Affiliations:** 1 MRC Population Health Research Unit, Nuffield Department of Population Health, University of Oxford, Oxford, UK; 2 Clinical Trial Service Unit & Epidemiological Studies Unit, Nuffield Department of Population Health, University of Oxford, Oxford, UK; 3 Faculty of Medicine, National Autonomous University of Mexico, Mexico City, Mexico; 4 Monterrey Institute of Technology and Higher Education, Nuevo Leon, Mexico; 5 Experimental Research Unit from the Faculty of Medicine, National Autonomous University of Mexico, Mexico City, Mexico

**Keywords:** Diabetes Mellitus, Type 2, Infections, Cohort Studies

## Abstract

**Introduction:**

Although higher risks of infectious diseases among individuals with diabetes have long been recognized, the magnitude of these risks is poorly described, particularly in lower income settings. This study sought to assess the risk of death from infection associated with diabetes in Mexico.

**Research design and methods:**

Between 1998 and 2004, a total of 159 755 adults ≥35 years were recruited from Mexico City and followed up until January 2021 for cause-specific mortality. Cox regression yielded adjusted rate ratios (RR) for death due to infection associated with previously diagnosed and undiagnosed (HbA1c ≥6.5%) diabetes and, among participants with previously diagnosed diabetes, with duration of diabetes and with HbA1c.

**Results:**

Among 130 997 participants aged 35–74 and without other prior chronic diseases at recruitment, 12.3% had previously diagnosed diabetes, with a mean (SD) HbA1c of 9.1% (2.5%), and 4.9% had undiagnosed diabetes. During 2.1 million person-years of follow-up, 2030 deaths due to infectious causes were recorded at ages 35–74. Previously diagnosed diabetes was associated with an RR for death from infection of 4.48 (95% CI 4.05–4.95), compared with participants without diabetes, with notably strong associations with death from urinary tract (9.68 (7.07–13.3)) and skin, bone and connective tissue (9.19 (5.92–14.3)) infections and septicemia (8.37 (5.97–11.7)). In those with previously diagnosed diabetes, longer diabetes duration (1.03 (1.02–1.05) per 1 year) and higher HbA1c (1.12 (1.08–1.15) per 1.0%) were independently associated with higher risk of death due to infection. Even among participants with undiagnosed diabetes, the risk of death due to infection was nearly treble the risk of those without diabetes (2.69 (2.31–3.13)).

**Conclusions:**

In this study of Mexican adults, diabetes was common, frequently poorly controlled, and associated with much higher risks of death due to infection than observed previously, accounting for approximately one-third of all premature mortality due to infection.

**WHAT IS ALREADY KNOWN ON THIS TOPIC**

- Studies in high-income countries suggest diabetes is associated with an approximate doubling of the risk of infection-related mortality, but risks are poorly characterized in lower income settings.

**WHAT THIS STUDY ADDS**

- In this prospective study of 150 000 Mexican adults, previously diagnosed diabetes was associated with fourfold higher risk of death from infection, and up to 10-fold higher risk of death due to specific infectious causes.
- Relative risks were higher with longer diabetes duration and worse glycemic control.

**HOW THIS STUDY MIGHT AFFECT RESEARCH, PRACTICE OR POLICY**

- Prevention of diabetes and management of infectious disease risks among individuals with diabetes could substantially reduce premature death due to infectious causes among adults in Mexico.

## Introduction

Despite the classic dichotomy of communicable and non-communicable diseases, clear inter-relationships exist between them, as demonstrated by the COVID-19 pandemic.^1^ Greater susceptibility to infections has long been recognized in diabetes. However, infection has recently been described as an ‘emerging’ complication of the condition,^2^ and has received comparably little attention in clinical guidelines and practice, with prevention efforts consisting of, at best, vaccination (eg, influenza and pneumococcal vaccinations^3^) and foot care.^4^

The limited focus on infectious complications of diabetes likely in part reflects the availability of only limited, and frequently inconsistent, population-based evidence on the relevance of diabetes for infectious disease risks. Studies in high-income countries suggest an approximate doubling of the risk of infection-related death in diabetes.^5–8^ However, findings from lower income settings are more varied. Risks among East Asian populations appear similar to those in high-income countries,^9 10^ while previous findings from Latin America suggest up to sixfold higher risks of death from infectious causes in diabetes.^11 12^ These differences may reflect differences in the typical characteristics of diabetes; for example, in levels of glycemic control, which may drive the risk of infectious diseases.^13^ However, inconsistent findings from frequently underpowered observational studies with inadequate control for confounding limit our understanding of this,^13^ with scarce evidence available from trials.^14^ The relevance of diabetes-related comorbidities is similarly unclear; although early studies suggested macrovascular complications mediate the association of diabetes with mortality from infectious causes,^15^ subsequent studies have varied in their assessment of the relevance of vascular diseases.^16 17^ Furthermore, few studies have examined the full range of infectious diseases, with many, particularly in lower income settings, focusing only on one or a subset of infections. Thus, considerable uncertainty persists regarding the relevance of diabetes for infectious disease mortality and factors influencing this, particularly in low and middle-income country settings.

Using data from the Mexico City Prospective Study of approximately 150 000 adults, we report on the associations of diabetes with risk of death from infectious causes, exploring how duration of diabetes diagnosis and glycemic control influence these risks.

## Research design and methods

### Study population

Details of the Mexico City Prospective Study design, methods and population have been reported previously.^18^ Briefly, between 1998 and 2004, households within two urban districts of Mexico City (Coyoacán and Iztapalapa) were visited and all residents aged 35 years or older were invited to participate.

### Data collection

Trained nurses administered electronic questionnaires in participants’ houses, collecting information on sociodemographic status, lifestyle factors (eg, smoking and alcohol consumption) and personal medical history, including current medication. Physical measurements were undertaken using calibrated instruments, including height, weight, hip and waist circumferences and sitting blood pressure. A 10 mL non-fasting venous blood sample was collected into an EDTA vacutainer and separated into two plasma and one buffy coat aliquots for long-term storage at −150°C. HbA1c levels were measured in buffy coat samples using a validated high-performance liquid chromatography method^11^ on HA-8180 analyzers with calibrators traceable to International Federation of Clinical Chemistry standards.^19^

### Assessment of glycemic status

Participants who reported at recruitment to have been previously diagnosed with diabetes by a doctor, or who reported taking one or more medications for diabetes were defined as having previously diagnosed diabetes. These participants provided information on their approximate date of diagnosis. Among those without previously diagnosed diabetes, undiagnosed diabetes was defined as an HbA1c level of 6.5% (equivalent to 48 mmol/mol) or higher, and pre-diabetes was defined as an HbA1c level between 6.0% and 6.4% (equivalent to 42–47 mmol/mol).^20^ Normoglycemia was defined as HbA1c <6.0%. Participants with diabetes diagnosed before 35 years and taking insulin at recruitment were considered to have likely type 1 diabetes.

### Follow-up for mortality

Information on cause of death is obtained through probabilistic linkage (based on name, age and sex) to the Mexican electronic death registry. All diseases recorded on death certificates are coded using the International Statistical Classification of Diseases and Related Health Problems, Tenth Revision. Deaths are reviewed by study clinicians who, where necessary, recode the underlying cause of death.^11^ Participant deaths for the present study were tracked until 1 January 2021. The primary endpoints in this paper are deaths for which any infection was recorded as the underlying cause, and six main subcategories of respiratory, urinary tract, gastrointestinal, and skin, bone and connective tissue infections, septicemia, and other infections (online supplemental table S1).

10.1136/bmjdrc-2022-003199.supp1 Supplementary data

### Statistical analysis

Analyses excluded participants aged 85 years or older, with previously diagnosed chronic diseases other than diabetes (ischemic heart disease, stroke, chronic kidney disease, cirrhosis, cancer, emphysema) or with likely type 1 diabetes. Those with missing or extreme exposure or covariate (see below) data, or who had an uncertain cause of death were also excluded.

Cox proportional hazards models, with time since entry into the study as the underlying timescale, were used to determine the relevance of previously diagnosed and undiagnosed diabetes and, among participants with previously diagnosed diabetes, of diabetes duration (<5, 5 to <10, or ≥10 years) and glycemic control (HbA1c <9.0%, 9.0% to <11.0%, or ≥11.0%) for infectious disease mortality, through estimation of cause-specific mortality rate ratios (RRs, estimated by the Cox hazard ratios). Among individuals with previously diagnosed diabetes, diabetes duration and HbA1c were subsequently examined as continuous variables once it was confirmed that there was no evidence against these associations being ‘log-linear’.^21^ Mortality RRs were stratified by age at risk (5-year groups) and sex, and adjusted for district (Coyoacán and Iztapalapa), educational level (university or college, high school, elementary school, other), smoking status (never, former, occasional, <10 cigarettes/day, ≥10 cigarettes/day), alcohol drinking (never, former, current), height (four equal groups), weight (four equal groups), waist circumference (four equal groups) and hip circumference (four equal groups). Analyses examining the relevance of diabetes duration were additionally standardized for glycemic control, and those examining glycemic control for diabetes duration (to the average levels of those with previously diagnosed diabetes). Group-specific variances were estimated (reflecting the amount of data in each glycemic status category), such that the RR for each category, including the reference category, is associated with a group-specific ('floated') 95% CI, enabling comparisons between any two categories and not only with the reference group.^22^ Participants who did not die due to the infectious disease of interest were censored at the earliest of death from any other cause, the end of the age-at-risk period of interest, or 31 December 2020. The main analyses examined premature mortality (ie, deaths before 75 years), but the relevance of previously diagnosed diabetes for infectious disease mortality was also examined at 75–84 years. Adjusted RRs were compared across strata of other covariates (sex, region, education, smoking, alcohol drinking and body mass index (BMI) quartiles). Additional analyses explored the relevance of pre-diabetes (HbA1c 6.0% to <6.5%) and of HbA1c levels within the normoglycemic range (<6.0%) for infectious disease mortality. Sensitivity analyses included participants with previously diagnosed chronic diseases other than diabetes at recruitment.

Assuming a causal relationship, the proportions of infectious disease deaths attributable to undiagnosed diabetes, previously diagnosed diabetes with HbA1c <9.0%, previously diagnosed diabetes with HbA1c ≥9.0%, and total diabetes (diagnosed and undiagnosed combined) were calculated for each group by (RR-1)/RR, where RR is the adjusted RR for infectious disease death for each group relative to participants without diabetes.

All analyses used SAS V.9.4. Figures were produced using R V.4.1.3.

## Results

### Participant characteristics

Of the 159 755 participants recruited, 20 379 were excluded from the present analyses. These comprised 2959 participants aged ≥85 years at recruitment, a further 7800 with prior chronic diseases other than diabetes, 229 with likely type 1 diabetes, 1869 with uncertain mortality linkage, and 7317 with missing or outlying data, and a further 205 participants who were recruited twice (data from the first visit at which a blood sample was collected were used for these participants). Among the remaining 139 376 participants, 130 997 were aged 35–74 at recruitment, and 8379 were aged 75–84 (online supplemental table S2).

Table 1 shows the baseline characteristics of the 130 997 participants aged 35–74 years at recruitment. Their mean (SD) age was 51 (10) years, 32% were men, and their mean BMI was 29.1 (4.8) kg/m^2^. Overall, 17.2% of participants had diabetes, including 12.3% (n=16 112) with previously diagnosed diabetes and 4.9% (n=6381) with undiagnosed diabetes. A further 5.3% (n=7008) of participants had an HbA1c level in the pre-diabetes range. Participants with diabetes at recruitment were older, less highly educated and less likely to be current smokers or alcohol drinkers than those without diabetes. The prevalence of previously diagnosed diabetes increased markedly with age from about 2% at 35–39 to over 25% at 70–74 (online supplemental figure S1). Diagnosis was, on average, 9 years prior to recruitment. Longer time since diagnosis was associated with a lower frequency of current smoking, and lower BMI and waist circumference, as well as with younger age at diagnosis. Most participants with previously diagnosed diabetes (79%) reported taking glucose-lowering medication, most commonly sulfonylureas (69%). However, the mean (SD) baseline HbA1c among participants with previously diagnosed diabetes was 9.1% (2.5%), and was higher among participants with a longer duration of diabetes, despite reportedly more frequent use of glucose-lowering medication.

**Table 1:** Baseline characteristics of 130 997 participants aged 35–74 years at recruitment

	No diabetes	Diabetes		Previously diagnosed diabetes by duration (years)			Previously diagnosed diabetes by HbA1c (%)			Overall
		Undiagnosed	Previously diagnosed	<5	5 to <10	≥10	<9	9 to <11	≥11	
Participants (n)	108 504	6381	16 112	4151	7809	4152	8021	4120	3971	130 997
Age, sex and socioeconomic factors										
Age (years)	49 (10)	54 (10)	58 (10)	55 (10)	57 (9)	62 (8)	59 (10)	57 (9)	55 (9)	51 (10)
Men, %	32	34	33	32	33	33	33	33	30	32
Resident of Coyoacán, %	41	33	34	27	35	41	35	34	33	40
Resident of Iztapalapa, %	59	67	66	73	65	59	65	66	67	60
University/college educated, %	18	10	8	8	9	6	8	8	6	16
Lifestyle factors, %										
Current smoker	30	27	24	27	25	19	22	25	25	29
Current alcohol drinker	70	66	59	60	61	53	56	60	62	68
Physical activity 1+ times/week	23	16	21	18	21	22	23	20	17	22
Anthropometry										
Height (cm)	156 (9)	155 (9)	154 (9)	155 (9)	155 (9)	154 (9)	155 (9)	155 (9)	154 (9)	156 (9)
Weight (kg)	71 (13)	76 (14)	70 (13)	73 (14)	70 (13)	66 (12)	71 (13)	70 (70)	67 (13)	71 (13)
BMI (kg/m^2^)	29.0 (4.7)	31.5 (5.2)	29.1 (5.0)	30.4 (5.2)	29.3 (4.9)	27.7 (4.6)	29.8 (4.9)	29.0 (4.9)	28.0 (5.1)	29.1 (4.8)
Waist circumference (cm)	93 (11)	101 (11)	97 (11)	98 (11)	97 (11)	94 (11)	98 (11)	97 (11)	94 (11)	94 (11)
Waist-to-hip ratio	0.89 (0.07)	0.93 (0.07)	0.92 (0.07)	0.93 (0.07)	0.92 (0.07)	0.93 (0.07)	0.93 (0.07)	0.93 (0.07)	0.92 (0.06)	0.90 (0.07)
HbA1c (%)	5.5 (0.4)	8.6 (2.1)	9.1 (2.5)	8.5 (2.5)	9.3 (2.4)	9.4 (2.4)	7.0 (1.1)	10.0 (0.6)	12.4 (1.2)	6.1 (1.7)
Duration of diabetes diagnosis (years)			9 (7)	3 (1)	8 (1)	19 (5)	9 (7)	10 (7)	10 (6)	
Age at diabetes diagnosis (years)			48 (10)	52 (10)	49 (9)	43 (9)	50 (10)	47 (10)	46 (9)	
Glucose-lowering medication, %										
Sulfonylurea			69	63	71	71	65	74	72	
Biguanide			18	13	19	22	17	20	20	
Insulin			6	2	5	11	4	8	7	
Other			1	2	1	1	1	1	2	
Any			79	70	81	86	75	85	82	

Mean (SD) or %.

BMI, body mass index.

### Diabetes and death from any infectious cause

**Figure 1:**
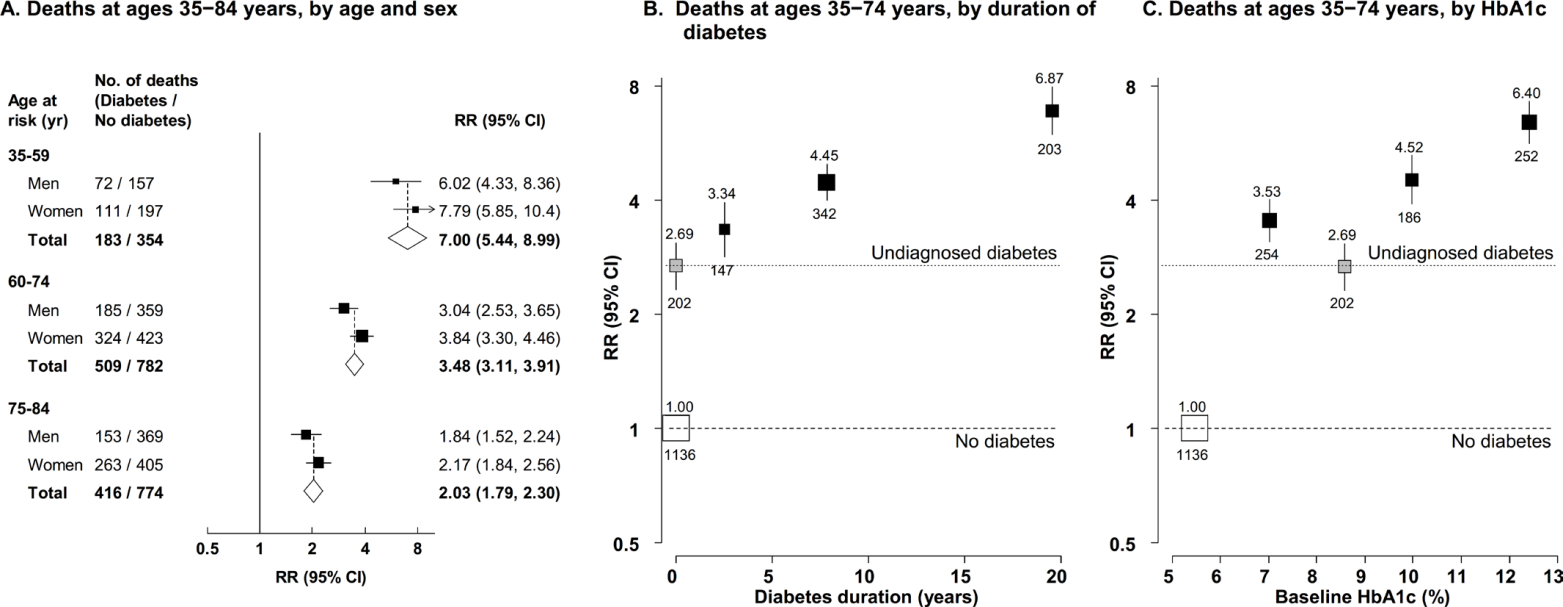
Relevance of previously diagnosed and undiagnosed diabetes to mortality from any infectious cause by age and sex (A), duration of diabetes (B), and glycemic control (C). (A) Mortality rate ratios (RRs) by age and sex for death from any infectious cause at ages 35–84 years for patients with previously diagnosed diabetes compared with those with no diabetes. Diamonds show values for men and women combined. The RRs for participants with undiagnosed diabetes compared with participants without diabetes were 4.21 (95% CI 3.10–5.72) at 35–59 years, 2.11 (95% CI 1.75–2.54) at 60–74 years, and 1.43 (95% CI 1.16–1.76) at 75–84 years. (B) Mortality RRs for death from any infectious cause by duration of diabetes at ages 35–74 years. (C) Mortality RRs for death from any infectious cause by glycemic control at ages 35–74 years. RRs in all panels are stratified by age at risk and sex (as appropriate), and adjusted for district, educational level, smoking status, alcohol drinking, height, weight, waist circumference and hip circumference. In (B) and (C), the RR estimates for those with previously diagnosed diabetes are also adjusted, respectively, for any HbA1c or diabetes duration differences between the groups (to the average HbA1c or duration seen for all those with previously diagnosed diabetes) in such a way that their information-weighted average equals the overall RR estimate for all those with previously diagnosed diabetes versus those with no diabetes. The numbers above the squares are the RRs and the numbers below the squares are the number of deaths in that group. In all panels, the size of each square is proportional to the amount of statistical information. Horizontal lines represent 95% CIs.

During approximately 2.1 million person-years (median 18 years) of follow-up, 2030 deaths due to infectious causes occurred at ages 35–74 (online supplemental table S1). There were 692 deaths (350 per 100 000 person-years) among participants with previously diagnosed diabetes, 202 (216 per 100 000 person-years) among participants with undiagnosed diabetes, and 1136 (63 per 100 000 person-years) among those without diabetes. These included 1198 deaths from respiratory infection, 211 from urinary tract infection, 176 from septicemia, 201 from gastrointestinal infection, and 109 from skin, bone or connective tissue infection. Among all 139 376 participants aged 35–84 at recruitment, there were 1292 deaths (491 per 100 000 person-years) due to any infectious cause at ages 75–84. Figure 1A shows age-specific and sex-specific RRs for death from any infectious cause among participants with previously diagnosed diabetes versus those without diabetes at recruitment. RRs were stronger at younger ages: 7.00 (95% CI 5.44–8.99) at 35–59, 3.48 (95% CI 3.11–3.91) at 60–74, and 2.03 (95% CI 1.79–2.30) at 75–84. At all ages, RRs did not differ much between men and women.

Previously diagnosed diabetes of less than 5 years’ duration was associated with an RR for death from any infectious cause at ages 35–74 years of 3.34 (95% CI 2.80–3.98), while participants with 5–10 years and ≥10 years’ duration had RRs of 4.45 (95% CI 3.93–5.05) and 6.87 (95% CI 5.86–8.06), respectively (figure 1B). Among participants with previously diagnosed diabetes, each additional year of diagnosed diabetes was associated with 3% (RR 1.03 (95% CI 1.02–1.05)) higher risk of death. Compared with participants without diabetes, the RR for death from any infectious cause at ages 35–74 years among those with undiagnosed diabetes was 2.69 (95% CI 2.31–3.13). Higher baseline HbA1c among participants with previously diagnosed diabetes was associated with higher infection-related death RRs (figure 1C). Compared with participants without diabetes, those with previously diagnosed diabetes with a baseline HbA1c <9.0% had an RR for death from any infectious cause at ages 35–74 years of 3.53 (95% CI 3.06–4.07), for those with a baseline HbA1c of 9.0% to <11.0% the death RR was 4.52 (95% CI 3.86–5.30), and for those with a baseline HbA1c of ≥11.0% it was 6.40 (95% CI 5.56–7.38). In those with previously diagnosed diabetes, each 1.0% higher baseline HbA1c was associated with 12% (RR 1.12 (95% CI 1.08–1.15)) higher risk of death from any infectious cause. For deaths at ages 35–74 years, previously diagnosed diabetes was more strongly associated with death due to any infectious cause among those with a lower BMI level (who had a higher HbA1c level), but RRs otherwise differed little by baseline characteristics (online supplemental figure S2).

### Diabetes and death from specific infectious causes

**Figure 2:**
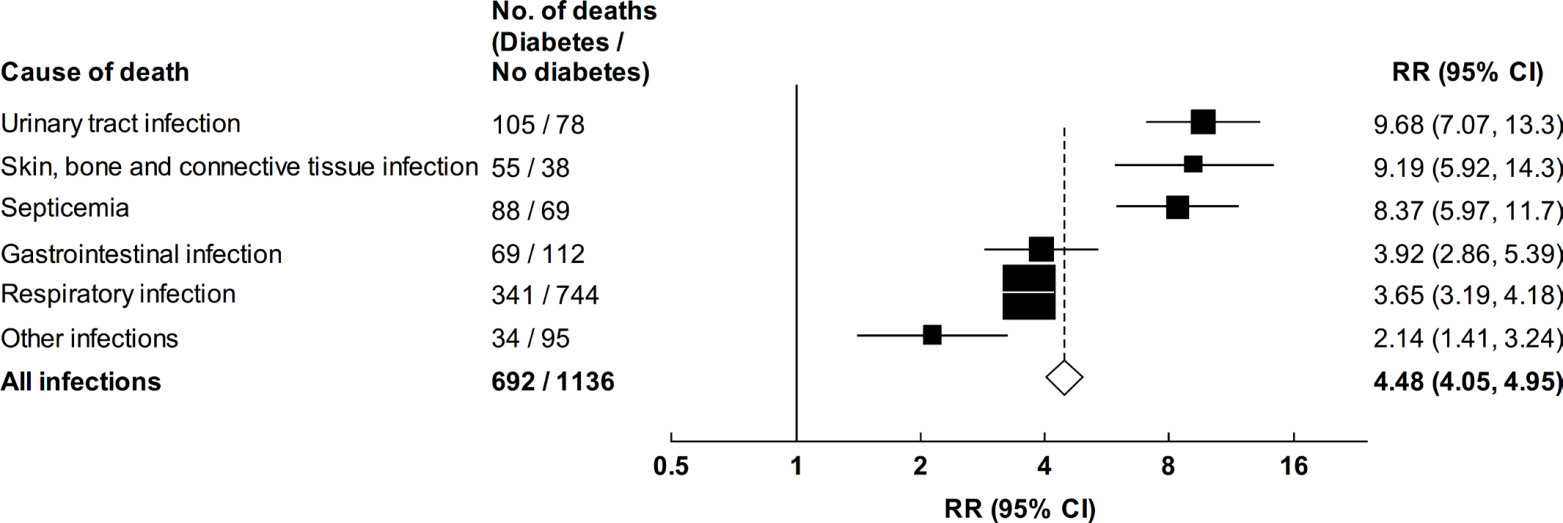
Relevance of previously diagnosed diabetes to mortality from infectious causes at ages 35–74 years. Mortality rate ratios (RRs) for deaths due to infectious causes at ages 35–74 years for patients with previously diagnosed diabetes versus those with no diabetes. RRs are stratified by age at risk and sex, and adjusted for district, educational level, smoking status, alcohol drinking, height, weight, waist circumference and hip circumference. The RR for death due to pneumonia was 5.32 (95% CI 4.47–6.35), and for death due to COVID-19 was 1.76 (95% CI 1.30–2.37). The RRs for participants with undiagnosed diabetes compared with participants without diabetes were 5.67 (95% CI 3.63–8.85) for urinary tract infection deaths, 5.43 (95% CI 2.98–9.90) for skin, bone and connective tissue infection deaths, 4.26 (95% CI 2.53–7.17) for septicemia deaths, 2.36 (95% CI 1.45–3.83) for gastrointestinal infection deaths, 2.35 (95% CI 1.92–2.87) for respiratory infection deaths, 0.99 (95% CI 0.43–2.28) for deaths due to other infections, and 2.69 (95% CI 2.31–3.13) for all infectious disease deaths. The size of each square is proportional to the amount of data available, and the unshaded diamond represents the values for mortality from any infectious cause. Horizontal lines represent 95% CIs.

**Figure 3:**
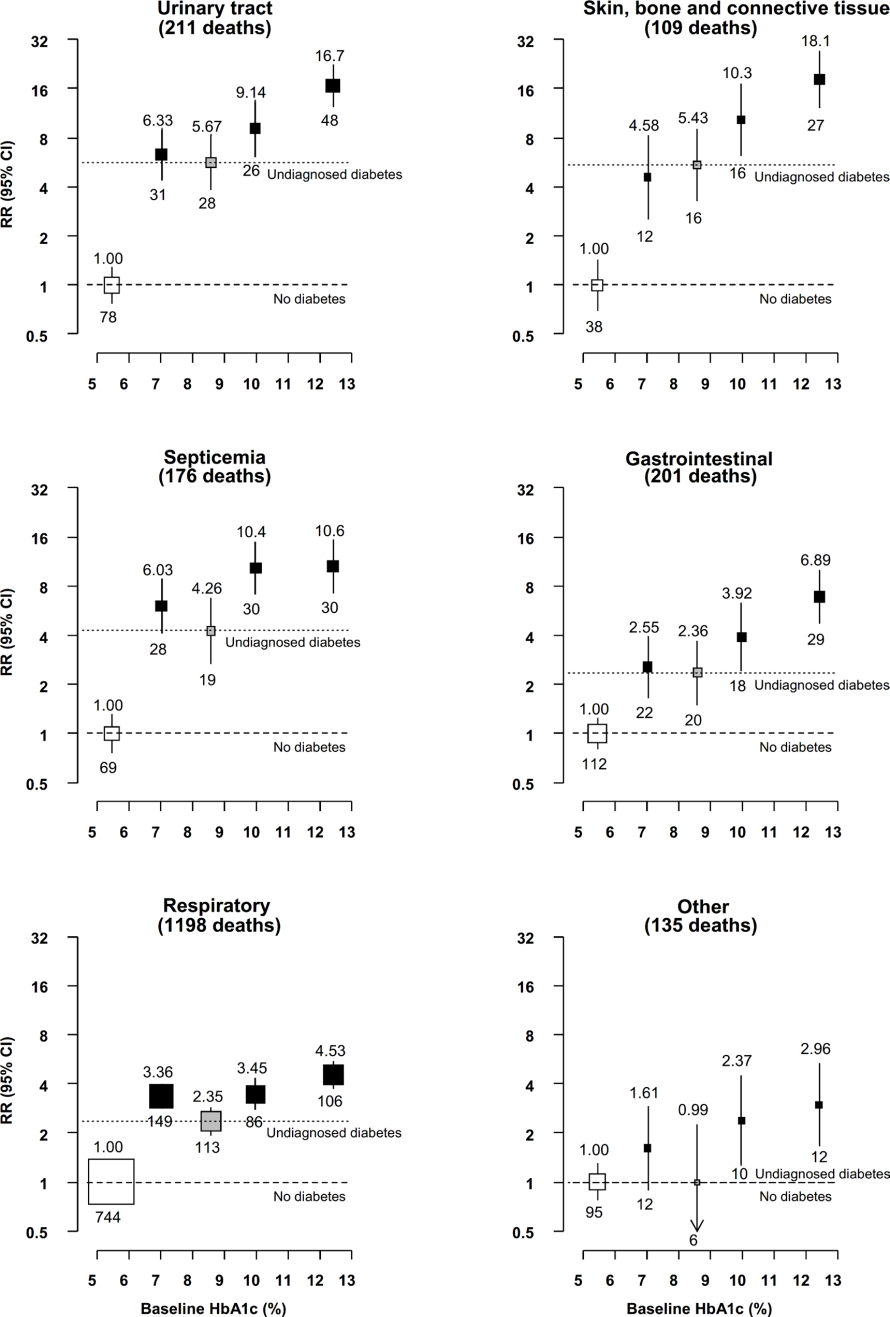
Relevance of previously diagnosed and undiagnosed diabetes to mortality from infectious causes at ages 35–74 years by glycemic control. Rate ratios (RR) are stratified by age at risk and sex, and adjusted for district, educational level, smoking status, alcohol drinking, height, weight, waist circumference and hip circumference. Analyses are additionally adjusted for duration of diabetes diagnosis among participants with previously diagnosed diabetes. Unfilled squares represent no diabetes. Gray squares represent undiagnosed diabetes. Black squares represent previously diagnosed diabetes. The numbers above the squares are the RRs and the numbers below the squares are the number of deaths in that group. The size of each square is proportional to the amount of data available. The error bars represent 95% CIs.

Figure 2 presents RRs for death at ages 35–74 years from specific infectious causes among participants with previously diagnosed diabetes compared with those without diabetes. There were notably strong associations with death from urinary tract infection (RR 9.68 (95% CI 7.07–13.3)), skin, bone and connective tissue infection (9.19 (95% CI 5.92–14.3)) and septicemia (8.37 (95% CI 5.97–11.7)). More modest but still substantial associations were observed for death from gastrointestinal (RR 3.92 (95% CI 2.86–5.39)) and respiratory (3.65 (95% CI 3.19–4.18)) infections. Deaths from respiratory infections largely comprised deaths from pneumonia (55%) and COVID-19 (36%), for which the mortality RRs were 5.32 (95% CI 4.47–6.35) and 1.76 (95% CI 1.30–2.37), respectively (4.86 (95% CI 2.87–8.23) and 1.69 (95% CI 1.25–2.27), respectively, when follow-up was limited to the period from 1 January to 31 December 2020). RRs for death from specific infectious diseases associated with previously diagnosed diabetes were similar in men and women (online supplemental figure S3), but were stronger at younger than at older ages (online supplemental figure S4). Undiagnosed diabetes was associated with more modest RRs for death from all specific infectious causes (figure 3). Analyses including participants with previously diagnosed chronic diseases at recruitment did not materially alter diabetes-associated RRs (data not shown).

Diabetes diagnosis duration was strongly positively associated with death from skin, bone and connective tissue infection and with death from septicemia (online supplemental figure S5). Participants with diagnosed diabetes of ≥10 years’ duration had an almost fourfold higher death rate due to skin, bone and connective tissue infection than participants with diagnosed diabetes of <5 years’ duration (RR 20.4 (floated 95% CI 12.9–32.4) vs 5.45 (floated 95% CI 2.83–10.5)). For death from septicemia, the RR (floated 95% CI) was approximately threefold higher after ≥10 years of diagnosed diabetes (RR 15.0 (10.4-21.8)) compared with <5 years (RR 5.38 (3.24–8.94)). By contrast, the increase in RR with longer diabetes durations was more modest for death from urinary tract infection (RR 15.2 (10.5–21.8) for diagnosed diabetes of ≥10 years vs 7.09 (4.57–11.0) for diagnosed diabetes <5 years’ duration) and with death from respiratory infection (5.28 (4.27–6.54) and 2.88 (2.32–3.58), respectively). Diagnosed diabetes duration was not clearly associated with risk of death from gastrointestinal infection.

Glycemic control among participants with previously diagnosed diabetes was strongly associated with death from skin, bone and connective tissue infection, with an almost fourfold higher rate of death among participants with baseline HbA1c ≥11.0% than among participants with baseline HbA1c <9.0%, reflecting death RRs of 18.1 (floated 95% CI 12.3–26.7) and 4.58 (floated 95% CI 2.59–8.10), respectively (figure 3). Moderately weaker (though still substantial) increases in the RR with higher HbA1c were observed for death from gastrointestinal infection (HbA1c <9.0%: RR 2.55 (1.68–3.89); HbA1c ≥11.0%: RR 6.89 (4.76–9.97)) and from urinary tract infection (HbA1c <9.0%: RR 6.33 (4.43–9.04); HbA1c ≥11.0%: RR 16.7 (12.5–22.3)). There were apparently positive, but more modest, associations between baseline HbA1c and death from septicemia and respiratory infection.

Assuming a causal relationship, approximately one-third of all infection deaths at ages 35–74 years were due to diabetes, including 18% attributable to uncontrolled previously diagnosed diabetes (HbA1c ≥9.0%), 9% to controlled previously diagnosed diabetes (HbA1c <9.0%) and 6% to undiagnosed diabetes (online supplemental table S3). This included more than half of deaths due to urinary tract infection, skin, bone and connective tissue infection and septicemia, with the greatest proportions accounted for by uncontrolled diagnosed diabetes.

### Non-diabetic glycemia and death from infectious causes

Pre-diabetes was associated with a modestly elevated mortality RR (1.38 (95% CI 1.18–1.58)) for death from any infectious cause when compared with baseline HbA1c levels <6.0%. However, there was no apparent association of HbA1c levels with risk of infectious disease mortality below this threshold (online supplemental figure S6). Higher HbA1c levels among participants without diabetes were associated with higher risks of death from septicemia, with RRs of 1.51 (95% CI 0.78–2.92), 1.65 (95% CI 0.78–3.47) and 2.31 (95% CI 0.94–5.66) at baseline HbA1c levels of 5.4% to <5.7%, 5.7% to <6.0% and 6.0% to <6.5%, respectively, compared with HbA1c <5.4% (online supplemental figure S7). A more modest positive association was observed with risk of death from respiratory infection (RRs of 1.11, 1.18 and 1.21, respectively). HbA1c levels among participants without diabetes were not related to mortality from other specific infectious causes studied.

## Conclusions

In this large prospective cohort of adults from Mexico City, previously diagnosed diabetes was associated with greater than fourfold higher risk of premature death from any infectious cause between 35 and 74 years of age. Death rates in diabetes were highest for urinary tract infection, skin, bone and connective tissue infection and septicemia, with up to 10-fold higher risks than participants without diabetes. Moreover, infectious disease mortality risks were higher among those with longer duration of diabetes or with higher baseline HbA1c levels. In this population with a high prevalence of frequently poorly controlled diabetes, the condition accounted for approximately one-third of all premature deaths due to infectious diseases.

The diabetes-associated risks of mortality due to infectious diseases observed in the present study are generally more extreme than those reported previously. Although infection-related mortality rates among patients with type 2 diabetes recruited from an outpatient clinic in Brazil were found to be six times higher than in the general adult population, this was based on a very small study population (n=471) and residual confounding may have explained some of the excess risk, since rates were standardized only for age and sex.^12^ In contrast, large-scale studies comprising populations from predominantly high-income countries have typically reported a more modest doubling of the risk of death due to infectious causes.^5 7 15 23^ For example, in the Emerging Risk Factors Collaboration’s individual participant data meta-analysis of 40 000 participants with previously or newly diagnosed diabetes and 675 000 participants without diabetes followed for an average of 14 years, diabetes was associated with a 2.4-fold higher risk of death due to all infections excluding pneumonia (n=1081) and a 1.7-fold higher risk of death due to pneumonia (n=2893).^5^ Differences between studies in the definitions of diabetes used may have contributed to differences in reported risks, as illustrated by the lower risks in undiagnosed, than diagnosed, diabetes in the present study. However, these would not explain the magnitude of differences observed, which could also reflect the influence of glycemic control or differing age distributions of study populations. Moreover, disparities between countries in access to healthcare interventions (eg, newer generation antibiotics, intensive care) with potential to differentially impact on population subgroups with greater susceptibility to severe infections, including individuals with diabetes,^13^ may have contributed to the comparably high diabetes-associated infectious disease mortality risks in this Mexican study population.

There is limited evidence available on the relevance of glycemic control for infectious disease mortality.^13 24^ Studies examining infectious disease outcomes more generally (ie, both fatal and non-fatal infections) have shown mixed findings, but many have found J-shaped or U-shaped associations of HbA1c levels with risk of both infectious disease incidence and mortality.^10 24–26^ For example, apparent J-shaped associations were observed between HbA1c levels and risks of both hospitalization for, and death from, infection among 85 000 patients with diabetes recorded in English primary care data, with lowest risks observed at levels of 6%–7%.^24^ In contrast, we found a strong positive association of baseline HbA1c levels with risk of death due to infectious causes among participants with previously diagnosed diabetes, with no apparent threshold in the association. The absence of higher risks at the lowest HbA1c levels in the present study likely reflects exclusion of participants with prior chronic diseases (in contrast with the other studies described), reducing potential for reverse causality and residual confounding, while the comparably clear positive association at higher HbA1c levels may reflect relatively poor glycemic control (mean HbA1c was 9.1% in the Mexico City study population, but 7.4% and 8.3% among participants with type 2 and type 1 diabetes, respectively, in the English study population^24^). Higher glucose concentrations may increase the risk of infectious diseases, as well as contributing to adverse outcomes following infection, through multiple mechanisms. These include impaired immune function, adversely impacting both humoral and cell-mediated immunity, and promotion of the growth of some microorganisms.^27^ However, trials of intensive glycemic control in diabetes have generally not investigated the impact on infections,^14^ and better evidence is needed to understand whether the association is causal. Evidence is also limited on the relevance of non-diabetic glycemia for infectious disease risk.^28^ Although we observed a higher risk of death due to any infectious cause among participants with pre-diabetes than among those with HbA1c levels in the truly ‘normoglycaemic’ range, this may simply reflect subsequent development of diabetes and associated infectious disease mortality risks in this group.

Duration of diagnosed diabetes showed a strong positive association with the risk of mortality due to infectious causes in the Mexico City population, independent of its association with glycemic control. This is consistent with the observed lower risks among individuals with undiagnosed, than with diagnosed, diabetes, despite similar mean HbA1c levels. Given the average 9-year duration of diabetes diagnosis, which is higher than in several previous studies,^10 29^ this may have contributed to the higher diabetes-associated risks of mortality due to infectious causes in this study population. Both longer diabetes duration^11^ and higher levels of glycemia^30 31^ are established risk factors for vascular complications of diabetes, which previous studies suggest may play a role in determining infectious disease risks and prognosis.^15 16^ In contrast, the associations presented herein differed little according to participants’ history of cardiovascular (ie, macrovascular) diseases. However, we were unable to account for macrovascular complications developed during follow-up in the study, or for microvascular complications. Few studies have simultaneously examined the association of diabetes with risks of death due to the full range of site-specific infections. However, this has been explored for infection-related outcomes more generally (including both primary care recorded diagnoses and hospitalizations),^10 16 27 29^ in many instances showing the highest diabetes-associated risks for the same, or closely related, infections as in the present study, although more modest than those observed in this Mexico City population.^16 27 29^ The notably strong associations of diabetes, glycemic control and diabetes duration with risk of death due to skin, bone and connective tissue infection may, at least in part, reflect the influence of chronic microvascular and macrovascular complications of diabetes, as well as the predominance of bacterial infections at these sites,^29^ and similar factors may explain the comparatively strong associations with death due to urinary tract infections and septicemia.

In the present study, we estimated that one-third of infectious disease deaths before age 75 could be attributed to diabetes. This clearly highlights the need for efforts to prevent infectious diseases among this Mexican population, particularly given the high prevalence of diabetes and poor glycemic control. Despite lower diabetes-associated relative mortality risks, respiratory infections accounted for the greatest proportion of infectious disease deaths among individuals with diabetes in the present study, highlighting the potential value of vaccination against respiratory pathogens in this population. Although pneumococcal and influenza vaccinations are recommended for all adults with diabetes in Mexico,^32^ uptake is reported to be low,^33^ but there is clear value in ensuring effective implementation of these existing vaccination guidelines.

Our study has certain limitations. First, diabetes may influence both the onset of infectious diseases and their course, and the focus on mortality prevented differentiation between these. However, mortality outcomes would be expected to be less susceptible to misclassification and to potential diagnostic biases, and readily permitted investigation of a wide spectrum of infectious diseases. Assessment of the relevance of glycemic control for infectious disease mortality risks was based on single HbA1c measurements, which may not reflect longer term trends. However, there is arguably clinical value in understanding the relevance of single measurements for future infectious disease mortality risks. More women than men were recruited into the study (because women were more likely to be at home when the fieldworkers’ visit was during standard working hours). However, the size of the study meant that large numbers of deaths were observed in both men and women, leading to reliable sex-specific estimates. The two study districts are also not representative of the overall Mexican population, or even the overall Mexico City population. However, prospective studies of non-representative cohorts of individuals can provide reliable evidence about the associations of risk factors with disease that are widely generalizable.^34 35^ Finally, the observational study design precludes assessment of the likely causality of the observed associations.

Diabetes is highly prevalent in Mexico and is associated with very high risks of infectious disease mortality, particularly among those with longer duration of diabetes diagnosis and poorer glycemic control. The findings presented clearly highlight the need for an increased focus on prevention of infectious diseases in the care of individuals with diabetes, including through effective implementation of existing vaccination policies, with potential for significant reductions in premature mortality. Moreover, prevention (or delay) of diabetes onset, including through prevention and management of the high levels of adiposity in the Mexican population, will be essential for reducing diabetes-associated infectious disease mortality.

10.1136/bmjdrc-2022-003199.supp2 Abstract translation This web only file has been produced by the BMJ Publishing Group from an electronic file supplied by the author(s) and has not been edited for content.

## Data Availability

Data are available upon reasonable request. Data from the Mexico City Prospective Study are available to bona fide researchers. For more details, the study’s Data and Sample Sharing policy may be downloaded (in English or Spanish) from https://www.ctsu.ox.ac.uk/research/mcps. Available study data can be examined in detail through the study’s Data Showcase, available at https://datashare.ndph.ox.ac.uk/mexico/.
