# Supplementary material for: Diabetes and infectious disease mortality in Mexico City
Source: BMJ Open Diabetes Res Care. 2023 Mar 8;11(2):e003199. doi: 10.1136/bmjdrc-2022-003199 (PMC10008442; doi:10.1136/bmjdrc-2022-003199)
Supplement: Supplementary data [file bmjdrc-2022-003199supp001.pdf]

BMJ Publishing Group Limited (BMJ) disclaims all liability and responsibility arising from any reliance placed on this supplemental material which has been supplied by the author(s)

Diabetes and infectious disease mortality in Mexico City

Online Supplemental Material, Table of Contents

**Supplementary tables**	**Page**
Table S1: Infectious disease endpoint ICD-10 codes and number of deaths at age-at-risk 35-74 years	2
Table S2: Baseline characteristics of 8379 participants aged 75-84 years at recruitment	3
Table S3: Infectious diseases mortality attributable to undiagnosed or previously-diagnosed diabetes at ages 35-74 years	4

**Supplementary figures**	
Figure S1: Prevalence of previously-diagnosed diabetes, by age	5
Figure S2: Relevance of previously-diagnosed diabetes to mortality from infectious causes at ages 35-74 years	6
Figure S3: Relevance of previously-diagnosed diabetes to mortality from infectious causes at ages 35-74 years, by sex	7
Figure S4: Relevance of previously-diagnosed diabetes to mortality from infectious causes at ages 35-84 years, by age	8
Figure S5: Relevance of previously-diagnosed and undiagnosed diabetes to mortality from infectious causes at ages 35–74 years, by duration of diabetes	9
Figure S6: Relevance of baseline HbA1c to mortality from any infectious cause among participants without previously-diagnosed diabetes at ages 35–74 years	10
Figure S7: Relevance of baseline HbA1c to mortality from infectious causes among participants without previously-diagnosed diabetes at ages 35–74 years	11

Table S1: Infectious disease endpoint ICD-10 codes and number of deaths at age-at-risk 35-74 years

Infectious disease endpoint	ICD-10 codes (number of such deaths recorded by 01 Jan 2021)
**Respiratory**	
Pneumonia	J12.9 (4), J15.1 (3), J15.7 (1), J15.9 (29), J18.0 (49), J18.1 (29), J18.2 (2), J18.8 (1), J18.9 (535)
COVID	U07.1 (225), U07.2 (203)
Other	A16.9 (2), B44.1 (1), J06.9 (1), J09 (2), J10.0 (2), J11.1 (1), J20.9 (3), J22.X (19), J34.8 (1), J44.0 (77), J44.1 (2), J85.0 (1), J85.2 (1), J86.9 (4)
**Urinary tract**	N10.X (1), N11.9 (1), N15.1 (10), N30.0 (1), N30.9 (1), N39.0 (190), N49.8 (6), N49.9 (1)
**Septicemia**	A41.5 (1), A41.9 (174), A48.3 (1)
**Gastrointestinal**	A04.7 (2), A06.0 (1), A09.0 (17), A09.9 (29), A09.X (11), B69.0 (2), K05.2 (1), K35.2 (4), K35.3 (3), K35.8 (4), K35.9 (2), K57.2 (2), K57.8 (7), K57.9 (10), K61.0 (3), K61.1 (1), K61.2 (1), K63.0 (1), K65.0 (19), K65.8 (1), K65.9 (50), K75.0 (12), K83.0 (17), K93.1 (1)
**Skin, bone and connective tissue**	Infections of the skin and subcutaneous tissue: L02.1 (2), L02.2 (6), L02.3 (2), L02.4 (1), L03.1 (4), L03.8 (1), L03.9 (4), L08.9 (30), L89.9 (6), L89.X (4), L90.5 (1), L98.4 (7)<br>Infections of bone and connective tissue: M00.9 (2), M60.0 (2), M72.5 (1), M72.6 (11), M79.8 (22), M86.9 (3)
**Other**	Tuberculosis: A16.2 (9), A16.5 (1), A17.0 (1), A18.0 (1), A18.1 (1), A18.2 (1), A18.3 (1), A19.9 (3)<br>Infections of central nervous system: A81.0 (2), A86.X (2), G00.9 (3), G03.9 (3), G04.2 (1), G04.9 (7), G06.0 (3)<br>Viral hepatitis: B16.9 (2), B17.1 (22), B18.1 (2), B18.2 (7), B18.9 (1), B19.0 (2), B19.9 (2),<br>HIV: B20.0 (1), B20.1 (2), B20.6 (2), B20.7 (3), B20.8 (7), B21.0 (1), B21.2 (1), B21.8 (1), B22.7 (2), B23.8 (4), B24.X (5)<br>Infections of circulatory system: I01.8 (1), I30.1 (1), I33.0 (6), I38.X (7),<br>Other: A49.8 (1), B46.5 (2), B90.9 (1), B94.8 (2), B99.X (1), H44.0 (1), J39.1 (1), N71.9 (1), N73.9 (2), N76.4 (1), Y40.5 (1)
**All**	All ICD-10 codes listed above

At ages 75-84 years there were 809 deaths from respiratory infection, 155 from urinary tract infection, 95 from septicemia, 134 f rom gastrointestinal infection, 72 from skin, bone and connective tissue infections and 27 deaths from other infections.

Table S2: Baseline characteristics of 8379 participants aged 75-84 years at recruitment

	No diabetes	Diabetes		Previously-diagnosed diabetes by duration, years			Previously-diagnosed diabetes by HbA1c, %			Overall
		Undiagnosed	Previously-diagnosed	<5	5 to <10	10+	<9	9 to <11	11+	
No. of participants	6026	548	1805	239	670	896	1236	359	210	8379
Age, sex and socioeconomic factors										
Age, years	79 (3)	78 (3)	78 (3)	78 (3)	78 (3)	78 (3)	79 (3)	78 (3)	78 (3)	79 (3)
Men	39	37	33	31	34	32	32	35	32	37
Resident of Coyoacán	35	28	31	24	31	32	31	26	34	34
Resident of Iztapalapa	65	72	69	76	69	68	69	74	66	66
University or college educated	4	4	3	3	3	3	3	2	2	4
Lifestyle factors										
Current smoker	13	13	9	11	9	9	9	11	9	12
Current alcohol drinker	51	46	43	44	45	41	42	47	41	49
Physical activity 1+ times/week	21	17	19	17	20	19	20	20	14	20
Anthropometry										
Height, cm	152 (9)	152 (9)	152 (9)	150 (9)	152 (9)	152 (9)	152 (9)	152 (9)	152 (9)	152 (9)
Weight, kg	64 (12)	68 (12)	64 (12)	65 (12)	66 (9)	63 (10)	64 (12)	65 (12)	62 (11)	64 (12)
BMI, kg/m^2	27.6 (4.6)	29.3 (4.6)	27.8 (4.6)	28.6 (4.5)	28.3 (4.5)	27.3 (4.5)	28.0 (4.6)	27.8 (4.4)	26.8 (4.2)	27.7 (4.6)
Waist circumference, cm	96 (11)	101 (10)	98 (11)	98 (11)	99 (11)	97 (11)	98 (11)	98 (11)	95 (11)	97 (11)
Waist-to-hip ratio	0.94 (0.07)	0.95 (0.07)	0.94 (0.07)	0.94 (0.07)	0.94 (0.07)	0.94 (0.07)	0.94 (0.07)	0.94 (0.07)	0.94 (0.07)	0.94 (0.07)
HbA1c, %	5.6 (0.4)	7.9 (1.8)	8.2 (2.1)	7.7 (2.1)	8.1 (2.2)	8.4 (2.1)	7.0 (1.0)	9.9 (0.6)	12.4 (1.3)	6.3 (1.6)
Duration of diabetes diagnosis, years			14 (9)	2 (1)	8(1)	22 (7)	13 (9)	15 (9)	15 (9)	
Age at diabetes diagnosis, years			64 (9)	76 (3)	70 (3)	57 (7)	65 (9)	63 (9)	63 (10)	
Glucose-lowering medication										
Sulphonylurea			69	61	73	69	68	69	75	
Biguanide			16	13	14	18	15	16	19	
Insulin			8	2	3	13	6	15	10	
Other			1	1	2	1	2	1	1	
Any			82	68	81	86	79	87	87	

Mean (SD) or %

Table S3: Infectious diseases mortality attributable to undiagnosed or previously-diagnosed diabetes at ages 35-74 years

Infectious disease	Number of deaths				Death RR (95% CI) vs. no diabetes				Attributable mortality, %			
	No diabetes	Undiagnosed diabetes	Previously-diagnosed diabetes		No diabetes	Undiagnosed diabetes	Previously-diagnosed diabetes		Undiagnosed diabetes	Previously-diagnosed diabetes		Total diabetes
			HbA1c <9.0%	HbA1c ≥9.0%			HbA1c <9.0%	HbA1c ≥9.0%		HbA1c <9.0%	HbA1c ≥9.0%	
Urinary tract infection	78	28	31	74	1.00	5.67	5.73	13.37	11	12	32	56
Skin, bone and connective tissue infection	38	16	12	43	1.00	5.43	3.86	14.70	12	8	37	57
Septicemia	69	19	28	60	1.00	4.26	5.38	11.16	8	13	31	52
Gastrointestinal infection	112	20	22	47	1.00	2.36	2.48	5.38	6	7	19	31
Respiratory infection	744	113	149	192	1.00	2.35	3.14	4.16	5	8	12	26
Other infections	95	6	12	22	1.00	0.99	1.50	2.77	0	3	10	13
**All infections**	**1136**	**202**	**254**	**438**	**1.00**	**2.69**	**3.25**	**5.69**	**6**	**9**	**18**	**33**

Rate ratios (RRs) are stratified by age-at-risk and sex, and adjusted for district, educational level, smoking status, alcohol drinking, height, weight, waist circumference and hip circumference. Attributable mortality calculated as number of deaths × (RR–1)/RR, where RR is the infectious disease death RR for that group relative to those without diabetes.

Figure S1: Prevalence of previously-diagnosed diabetes, by age

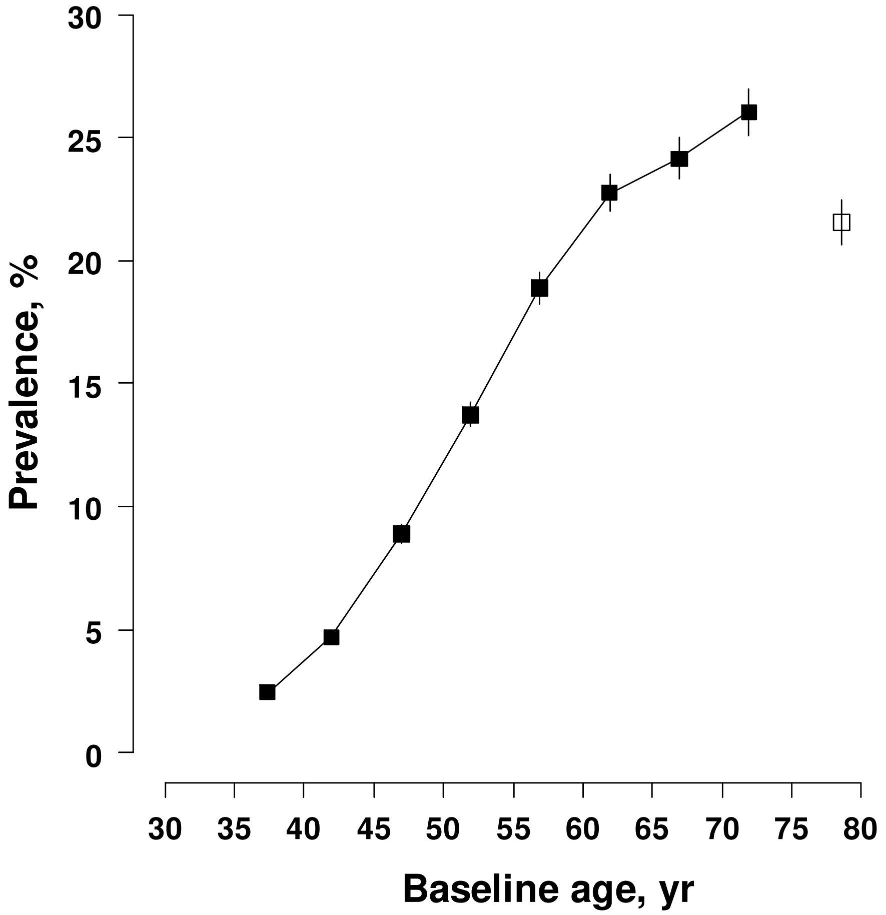

The unfilled square represents diabetes prevalence at ≥75 years. Error bars represent 95% confidence intervals.

Figure S2: Relevance of previously-diagnosed diabetes to mortality from infectious causes at ages 35-74 years

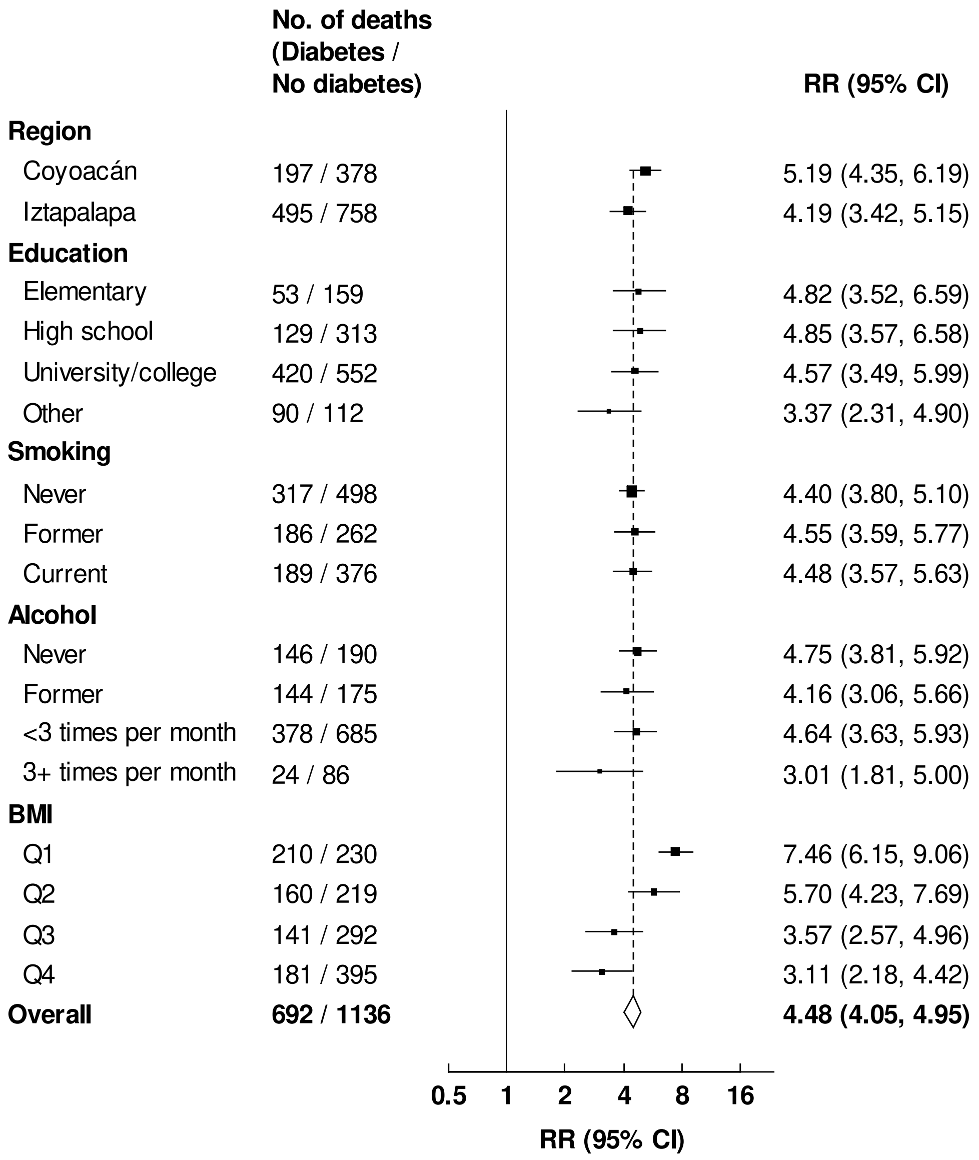

Mortality rate ratios (RRs) for deaths due to infectious diseases at ages 35–74 years, for patients with previously-diagnosed diabetes versus those with no diabetes. RRs are stratified by age-at-risk and sex, and adjusted for district, educational level, smoking status, alcohol drinking, height, weight, waist circumference and hip circumference. The size of each square is proportional to the amount of data available. Horizontal lines represent 95% confidence intervals.

Figure S3: Relevance of previously-diagnosed diabetes to mortality from infectious causes at ages 35-74 years, by sex

Cause of death	No. of deaths (Diabetes / No diabetes)	RR (95% CI)
**Urinary tract infection**		
Men	34 / 31	8.18 (4.98, 13.5)
Women	71 / 47	10.8 (5.53, 21.1)
**Skin, bone and connective tissue infection**		
Men	16 / 18	6.20 (3.12, 12.3)
Women	39 / 20	11.8 (4.73, 29.7)
**Septicemia**		
Men	38 / 21	12.0 (6.94, 20.6)
Women	50 / 48	6.67 (3.08, 14.5)
**Gastrointestinal infection**		
Men	23 / 43	3.74 (2.23, 6.26)
Women	46 / 69	3.98 (2.14, 7.44)
**Respiratory infection**		
Men	137 / 362	3.20 (2.61, 3.91)
Women	204 / 382	4.06 (3.16, 5.20)
**Other infections**		
Men	9 / 41	1.37 (0.66, 2.84)
Women	25 / 54	2.74 (1.35, 5.59)

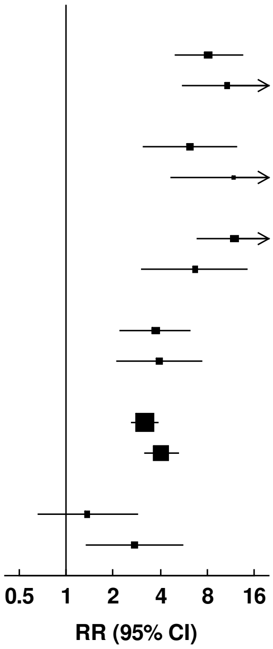

Mortality rate ratios (RRs) for deaths due to infectious diseases at ages 35–74 years, for patients with previously-diagnosed diabetes versus those with no diabetes. RRs are stratified by age-at-risk, and adjusted for district, educational level, smoking status, alcohol drinking, height, weight, waist circumference and hip circumference. The size of each square is proportional to the amount of data available. Horizontal lines represent 95% confidence intervals.

Bragg F, *et al. BMJ Open Diab Res Care* 2023; 11:e003199. doi: 10.1136/bmjdrc-2022-003199

Figure S4: Relevance of previously-diagnosed diabetes to mortality from infectious causes at ages 35-84 years, by age

Cause of death	Age at risk (yr)	No. of deaths (Diabetes / No diabetes)	RR (95% CI)
Urinary tract infection			
	35-59	32 / 18	26.1 (11.4, 59.3)
	60-74	73 / 60	6.14 (4.32, 8.74)
	75-84	71 / 71	3.58 (2.54, 5.07)
Skin, bone and connective tissue infection			
	35-59	18 / 10	22.4 (7.27, 69.1)
	60-74	37 / 28	6.50 (3.93, 10.8)
	75-84	25 / 39	2.29 (1.35, 3.87)
Septicemia			
	35-59	28 / 26	13.2 (6.53, 26.9)
	60-74	60 / 43	6.31 (4.22, 9.43)
	75-84	40 / 50	2.81 (1.81, 4.37)
Gastrointestinal infection			
	35-59	18 / 35	6.47 (2.96, 14.2)
	60-74	51 / 77	3.19 (2.22, 4.58)
	75-84	38 / 88	1.53 (1.03, 2.28)
Respiratory infection			
	35-59	77 / 222	4.82 (3.30, 7.04)
	60-74	264 / 522	2.91 (2.50, 3.39)
	75-84	238 / 506	1.84 (1.57, 2.16)
Other infections			
	35-59	10 / 43	3.06 (1.41, 6.64)
	60-74	24 / 52	1.93 (1.18, 3.16)
	75-84	4 / 20	0.67 (0.22, 2.01)

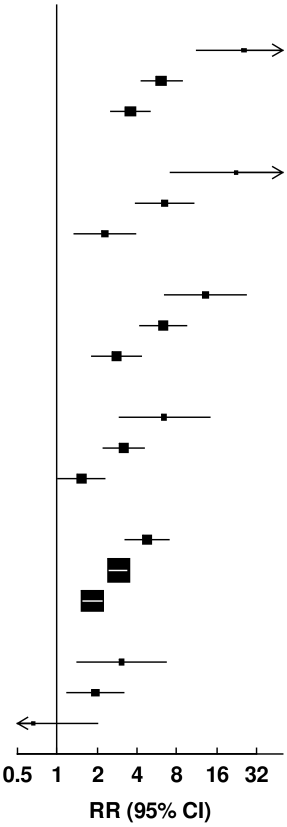

Mortality rate ratios (RRs) for deaths due to infectious diseases at ages 35-84 years, for patients with previously-diagnosed diabetes versus those with no diabetes. RRs are stratified by age-at-risk and sex, and adjusted for district, educational level, smoking status, alcohol drinking, height, weight, waist circumference and hip circumference. The size of each square is proportional to the amount of data available. Horizontal lines represent 95% confidence intervals.

Bragg F, *et al. BMJ Open Diab Res Care* 2023; 11:e003199. doi: 10.1136/bmjdrc-2022-003199

Figure S5: Relevance of previously-diagnosed and undiagnosed diabetes to mortality from infectious causes at ages 35–74 years, by duration of diabetes

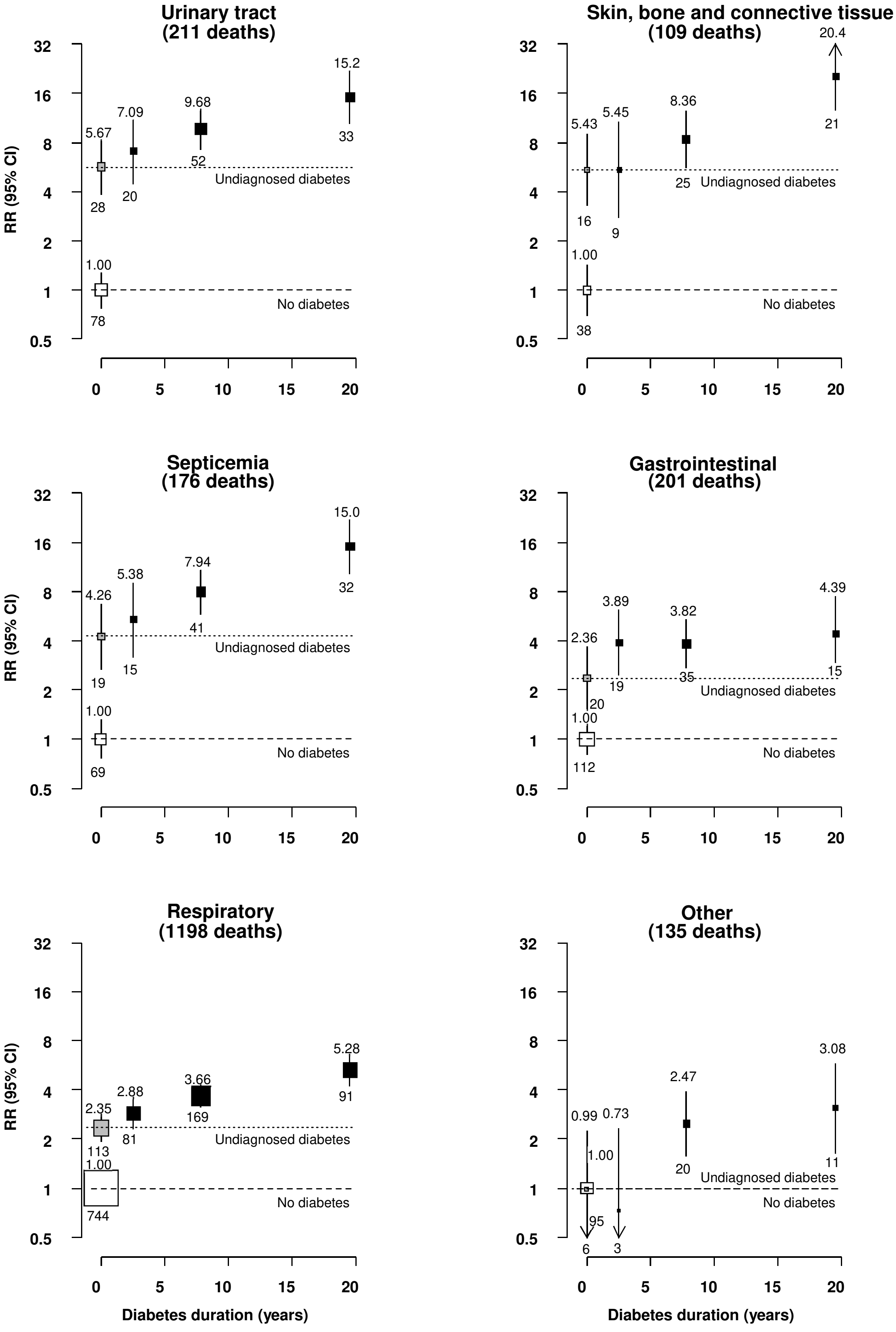

Rate ratios (RRs) are stratified by age-at-risk and sex, and adjusted for district, educational level, smoking status, alcohol drinking, height, weight, waist circumference and hip circumference. Analyses are additionally adjusted for HbA1c among participants with previously-diagnosed diabetes. Unfilled squares represent no diabetes. Grey squares represent undiagnosed diabetes. Black squares represent previously-diagnosed diabetes. The numbers above the squares are the RRs and the numbers below the squares are the number of deaths in that group. The size of each square is proportional to the amount of data available. The error bars represent 95% confidence intervals.

Figure S6: Relevance of baseline HbA1c to mortality from any infectious cause among participants without previously-diagnosed diabetes at ages 35–74 years

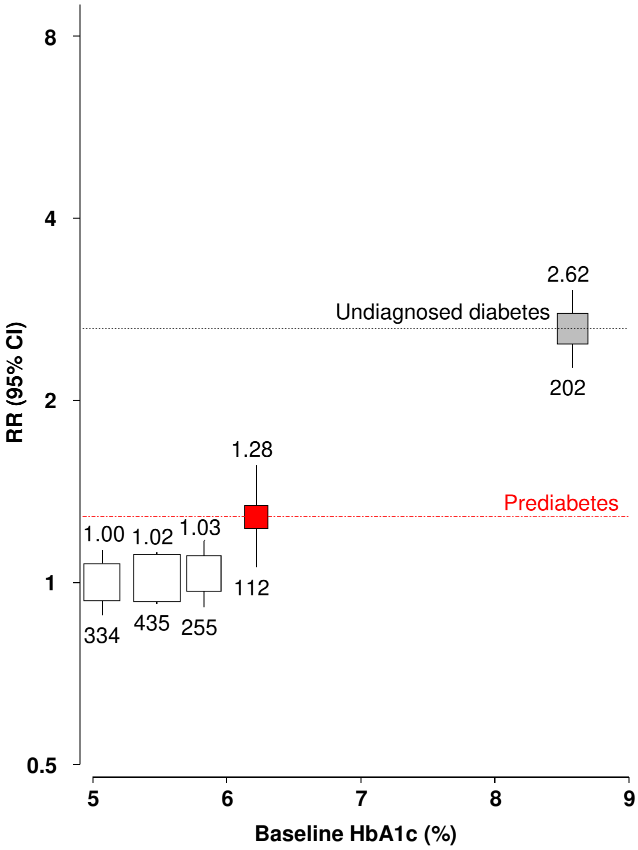

Rate ratios (RRs) are stratified by age-at-risk and sex, and adjusted for district, educational level, smoking status, alcohol drinking, height, weight, waist circumference and hip circumference. Unfilled squares represent no diabetes. Red squares represent prediabetes. Grey squares represent undiagnosed diabetes. The numbers above the squares are the RRs and the numbers below the squares are the number of deaths in that group. The size of each square is proportional to the amount of data available. The error bars represent 95% confidence intervals.

Bragg F, *et al. BMJ Open Diab Res Care* 2023; 11:e003199. doi: 10.1136/bmjdrc-2022-003199

Figure S7: Relevance of baseline HbA1c to mortality from infectious causes among participants without previously-diagnosed diabetes at ages 35–74 years

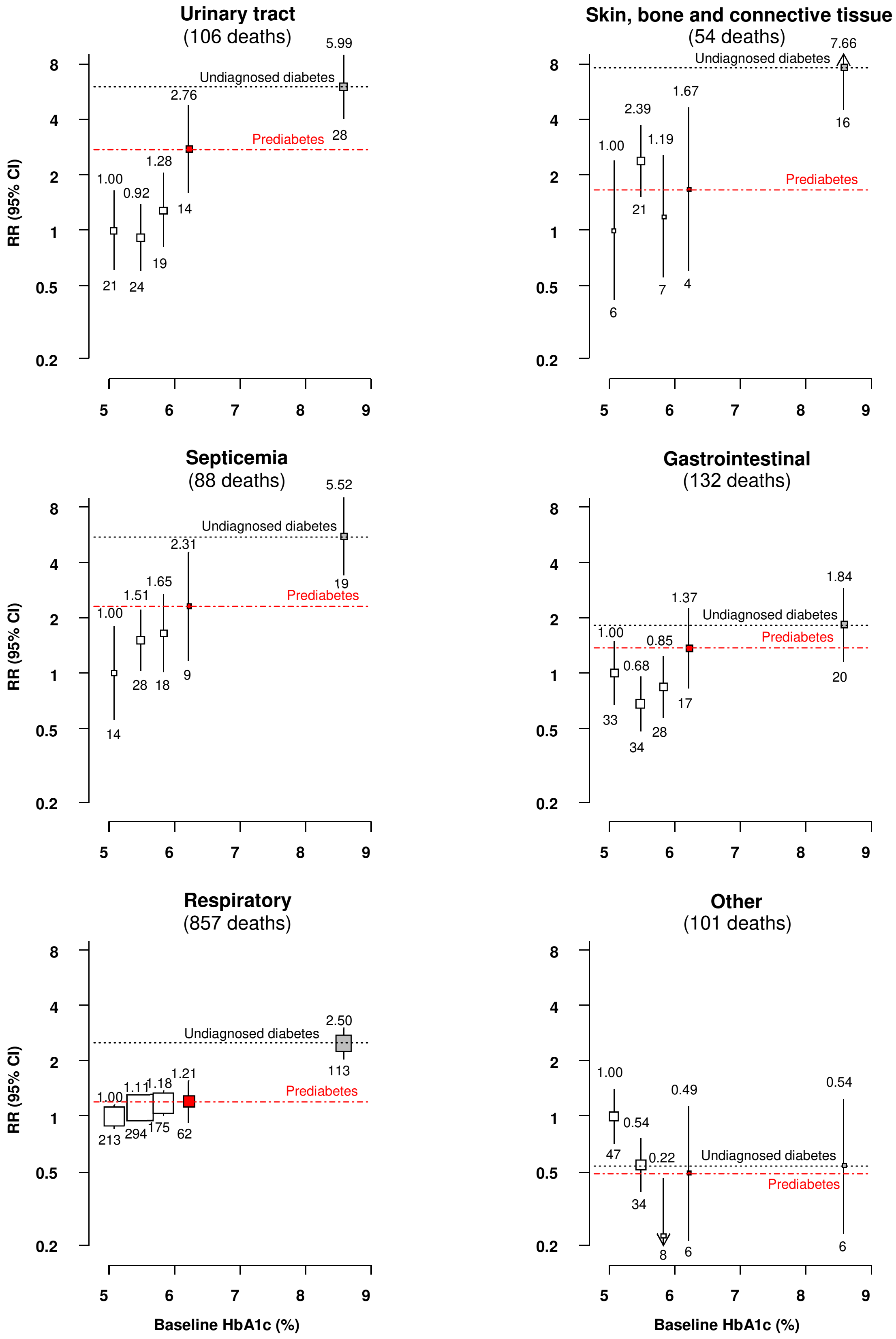

Rate ratios (RRs) are stratified by age-at-risk and sex, and adjusted for district, educational level, smoking status, alcohol drinking, height, weight, waist circumference and hip circumference. Unfilled squares represent no diabetes. Red squares represent prediabetes. Grey squares represent undiagnosed diabetes. The numbers above or below the squares are the RRs and the numbers below the squares are the number of deaths in that group. The size of each square is proportional to the amount of data available. The error bars represent 95% confidence intervals.

Bragg F, *et al. BMJ Open Diab Res Care* 2023; 11:e003199. doi: 10.1136/bmjdrc-2022-003199
