# Supplementary material for: Diabetes and infectious disease mortality in Mexico City
Source: BMJ Open Diabetes Res Care. 2023 Mar 8;11(2):e003199. doi: 10.1136/bmjdrc-2022-003199 (PMC10008442; doi:10.1136/bmjdrc-2022-003199)
Supplement: Abstract translation [file bmjdrc-2022-003199supp002.pdf]

Resumen

Introducción: No obstante que el riesgo elevado de enfermedades infecciosas en individuos con diabetes es conocido, la magnitud del incremento en este riesgo no ha sido definido de forma precisa, en especial en contextos de bajos recursos. Este estudio investigó el riesgo de muerte por infección asociado con diabetes en México.

Diseño de Investigación y Métodos: Entre 1998 y 2004, 159,755 adultos (≥35 años) fueron reclutados en de la Ciudad de México y han sido seguidos hasta enero 2021 para mortalidad por causa específica. Modelos de regresión de Cox proporcionaron razones de tazas (RR) de mortalidad por causas infecciosas asociadas con diabetes previamente diagnosticada y no diagnosticada (HbA1c ≥6.5%) y, en aquellos con diabetes previamente diagnosticada, con duración de diabetes y con HbA1c.

Resultados: De 130,997 participantes (edades 35-74) sin otras enfermedades crónicas al reclutamiento, 12.3% tenían diabetes previamente diagnosticada, con una HbA1c media (DE) de 9.1% (2.5%), y 4.9% tenían diabetes no diagnosticada. Durante 2.1 millones de años-persona de seguimiento, 2030 muertes por enfermedades infecciosas fueron registradas (edades 35-74). Diabetes previamente diagnosticada se asoció con un RR de mortalidad por infección de 4.48 (95% CI 4.05-4.95), comparado con no tener diabetes, con asociaciones notablemente fuertes con muerte por infección en tracto urinario (RR 9.68 [95% IC 7.07-13.3]) y en piel, hueso, y tejido conectivo (RR 9.19 [95% IC 5.92-14.3]), y por septicemia (RR 8.37 [95% IC 5.97-11.7]). En aquellos con diabetes previamente diagnosticada, mayor duración de diabetes (RR 1.03 [95% IC 1.02-1.05] por cada año) y mayor HbA1c (RR 1.12 [95% IC 1.08-1.15] por 1.0%) estuvieron independientemente asociados con mayor riesgo de mortalidad por infección. Aun en aquellos participantes con diabetes no diagnosticada, el riesgo de muerte por infección fue casi tres veces mayor que el riesgo en aquellos sin diabetes (RR 2.69 [95% IC 2.31-3.13]).

Conclusiones: En este estudio de adultos mexicanos, la diabetes es muy común, frecuentemente no está controlada, y se asocia con un incremento en el riesgo de mortalidad por infecciones que es mucho mayor al observado previamente, explicando aproximadamente un tercio de la mortalidad prematura por infecciones.
